# NOTICE OF DUPLICATE PUBLICATION: "Stability of meropenem in portable elastomeric infusion devices: which protocol should be implemented in clinical practice?"

**DOI:** 10.1128/spectrum.02063-23

**Published:** 2024-01-17

**Authors:** American Society for Microbiology

## NOTICE OF DUPLICATE PUBLICATION

The American Society for Microbiology (ASM) and *Microbiology Spectrum* are withdrawing the article mentioned below as it is an inadvertent duplicate publication:

Esteban-Cartelle B, Serrano DR, Pérez Menéndez-Conde C, Vicente-Oliveros N, Álvarez-Díaz A, Abete JF, Martín-Dávila P. 2024. Stability of meropenem in portable elastomeric infusion devices: which protocol should be implemented in clinical practice? Microbiol Spectr 12:e02063-23. https://doi.org/10.1128/spectrum.02063-23.

This article was accidentally published twice in the journal due to a technical error, and there are no ethical or integrity concerns regarding the original published article. The duplicate version of the article has been withdrawn to correct the scholarly record.

The original published article and its content are not affected by this withdrawal and can be found here: https://journals.asm.org/doi/10.1128/spectrum.02064-23

The American Society for Microbiology apologizes to the authors and its readers for the technical error and the inconvenience this may have caused. The authors agreed with the publication of this notice.

